# Light-Induced Conformational Alterations in Heliorhodopsin Triggered by the Retinal Excited State

**DOI:** 10.1021/acs.jpcb.1c04551

**Published:** 2021-08-03

**Authors:** Ishita Das, Alina Pushkarev, Mordechai Sheves

**Affiliations:** † Weizmann Institute of Science, Rehovot 7610001, Israel; ‡ Faculty of Biology, Technion—Israel Institute of Technology, Haifa 3200003, Israel

## Abstract

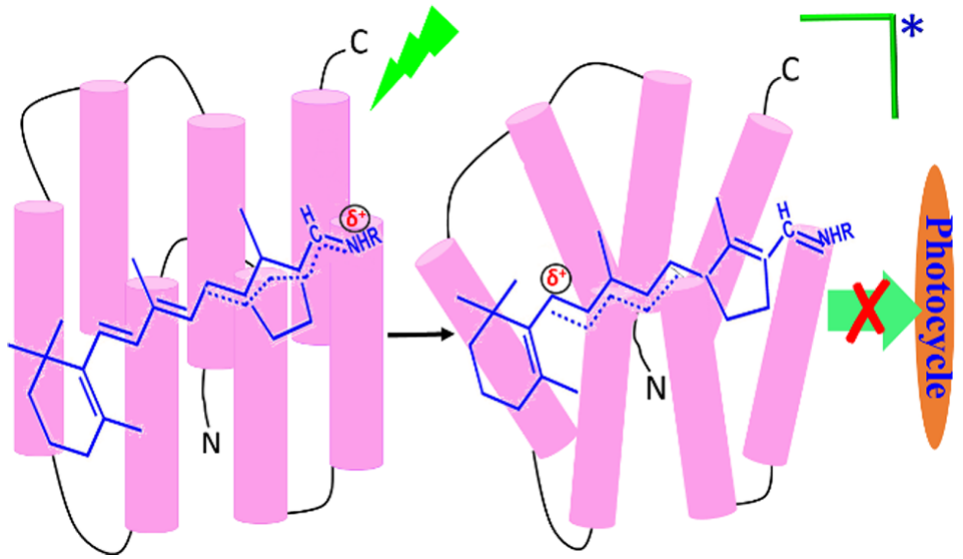

Heliorhodopsins are a recently discovered diverse retinal protein family with an inverted topology of the opsin where the retinal protonated Schiff base proton is facing the cell cytoplasmic side in contrast to type 1 rhodopsins. To explore whether light-induced retinal double-bond isomerization is a prerequisite for triggering protein conformational alterations, we utilized the retinal oxime formation reaction and thermal denaturation of a native heliorhodopsin of *Thermoplasmatales archaeon* SG8-52-1 (TaHeR) as well as a *trans*-locked retinal analogue (TaHeR_L_) in which the critical C_13_=C_14_ double-bond isomerization is prevented. We found that both reactions are light-accelerated not only in the native but also in the “locked” pigment despite lacking any isomerization. It is suggested that light-induced charge redistribution in the retinal excited state polarizes the protein and triggers protein conformational perturbations that thermally decay in microseconds. The extracted activation energy and the frequency factor for both the reactions reveal that the light enhancement of TaHeR differs distinctly from the earlier studied type 1 microbial rhodopsins.

## Introduction

Microbial and animal rhodopsin protein (type 1 and type 2, respectively) families are both composed of seven transmembrane α-helices with the C-terminus facing inside of the cell.^1^ The proteins contain a retinal chromophore covalently bound to a lysine residue via a protonated Schiff base bond. In microbial rhodopsins, the retinal chromophore undergoes light-induced double-bond isomerization from an all-*trans* to 13-*cis* isomer followed by a sequence of protein conformational alterations.^1−4^

In spite of the well-accepted fact that relevant protein structural changes are associated with photointermediates formed following light absorption, it was proposed that light-induced protein conformational changes can occur even in the absence of the retinal double-bond isomerization process. The proposal was based on studies with bacteriorhodopsin using atomic force sensing (AFS) methodology and electron paramagnetic resonance (EPR) spectroscopy of apo-bacteriorhodopsin membrane hosting synthetic nonisomerizable chromophores.^5−7^ Light acceleration of the EPR-probe redox reaction indicated that the protein experiences structural changes even without retinal double-bond isomerization.^5^ Retinal protonated Schiff base reaction with hydroxylamine (HA) (a nonphotochemical reaction) has been used as a sensitive method to detect light-induced protein conformational alterations in a few type 1 microbial rhodopsins.^8−11^

Recently, a new retinal protein family was discovered, named heliorhodopsin (HeR), which distantly relates to type 1 rhodopsins and undergoes all-*trans* to *cis* retinal photoisomerization.^12^ However, the residues comprising the retinal pocket, especially the hydrophobic ones, are divergent. The overall sequence identity of the HeR derived from *Thermoplasmatales archaeon* SG8-52-1 (TaHeR), which is employed in the current study, is only ∼8% of the type 1 microbial rhodopsin family.^13^ Considering its long-lifetime photocycle, it has been suggested that HeR acts as a signaling photoreceptor, though its exact function is not clear yet.^12^ HeR possesses a unique topology in which the protein N-termini faces the cell cytoplasm, an inverted orientation relative to both type 1 and type 2 rhodopsins. This unique topology and a very dissimilar residue sequence raise the question whether light-induced protein conformational alterations can occur in TaHeR without the retinal double-bond isomerization. A recent study with resonance Raman spectroscopy suggested a discrete geometry of the retinal protonated Schiff base owing to structural differences of the retinal pocket in HeR.^14^ Considering this distinguished structural characteristic of the HeRs as compared to type 1 family, we have explored in the present study the effect of retinal light absorption on the protein conformation in HeR. We aimed at detecting whether light-induced protein conformation changes can occur without retinal double-bond isomerization even when HeR has these geometrical, structural, and sequence differences from other retinal proteins.

## Experimental Methods

### Expression and Purification of TaHeR

The gene encoding *T. archaeon* HeR (GenBank ID: KYK26602.1) was synthesized (Genscript) and subcloned into the pET21a (+)-vector with an N-terminal 6×His tag as reported previously.^1^ Proteins were expressed in *Escherichia coli* strain C43 (DE3). The cells were grown in LB medium with 100 μg/mL ampicillin at 37 °C to an optical density (OD_660_) of 0.5–0.6. Overexpression was induced by 1 mM isopropyl β-d-thiogalactopyranoside (IPTG) for 12 h. All-*trans* retinal was added to achieve a final concentration of 10 μM and kept for an additional 4 h at the same temperature. The cells were harvested by centrifugation (15 min, 7000 rpm, 4°C). The collected cells were resuspended in buffer S (50 mM MeS, 300 mM NaCl, 5 mM imidazole, 5 mM MgCl_2_; pH 6.5) containing 1% (w/v) *n*-dodecyl-β-d-maltoside (DDM) and lysed with lysozyme (0.1 mg/mL) overnight at 4°C in the presence of DNase and a protease inhibitor. The protein, solubilized in DDM, was isolated by centrifugation (45 min, 18 000 rpm, 4 °C) and then loaded on a Co^2+^-NTA resin (Thermo Fisher Scientific) material preequilibrated with buffer S (50 mM 2-(*N*-morpholino)ethanesulfonic acid, MES, 300 mM NaCl, 5 mM Imidazole, pH 6.5). Unspecific bound proteins were removed by washing with buffer W (50 mM MES, 300 mM NaCl, 50 mM imidazole, 0.06% DDM; pH 6.5). His-tagged proteins were eluted with buffer E (50 mM MES, 300 mM NaCl, 500 mM imidazole, 0.06% DDM; pH 6.0). Imidazole was removed from the desired protein by dialysis against buffer D (20 mM HEPES, 500 mM NaCl, 0.05% (w/v) DDM; pH 7.0).

### Apoprotein Preparation and Reconstitution with *trans*-Locked Retinal

Apoproteins were prepared by the removal of all-*trans* retinal from the wild-type TaHeR by the formation of retinal oxime with hydroxylamine at pH 7 under light irradiation with a Schott 250 W cold light source (with a long-pass (>500 nm) cutoff filter). The excess hydroxylamine was removed by washing the sample thrice with 0.02% DDM solution containing 200 mM NaCl in a Centricon filter (15 000 MWCO). To reconstitute the apoprotein with the retinal analogue, the apoprotein was incubated with 1.5 equivalent of the *trans*-locked retinal in 0.06% DDM containing 300 mM NaCl at pH 7 and 25 °C for 2 days.

### Hydroxylamine Reaction and Thermal Decomposition with Native and Artificial Pigments

All of the experiments were carried out at pH 7. The UV–vis absorption spectral measurements were taken with a Cary 8454 UV–vis spectrophotometer (Agilent Technologies, CA) equipped with a thermostated cuvette holder (Agilent 89090A). All of the light reactions at various temperatures were performed under an identical irradiation condition with a Schott 250 W cold light source (with a long-pass (>520 nm) cutoff filter). The temperature fluctuated by ±0.5 °C.

The concentration of hydroxylamine used (0.5 M) in the reactions was considerably high relative to the protein concentration (which was in the range of 1.5–2 μM); thus, the reaction can be assumed to be of first order with respect to the pigment. Therefore, the kinetic traces were followed at the pigment absorption maxima and fitted to single-exponential or biexponential decay components by the following equation
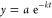
1or
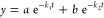
2

where *y* is the pigment remaining, *k* is the rate of the reaction, and *a* and *b* are the coefficients related to the relative amount of each component. To determine the activation energy (*E*_a_) and the frequency factor (*A*) of the processes, the obtained reaction rates in the dark and in light at various temperatures (*T*) were fitted to the Arrhenius equation.
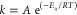
3

## Results and Discussion

**Figure 1:**
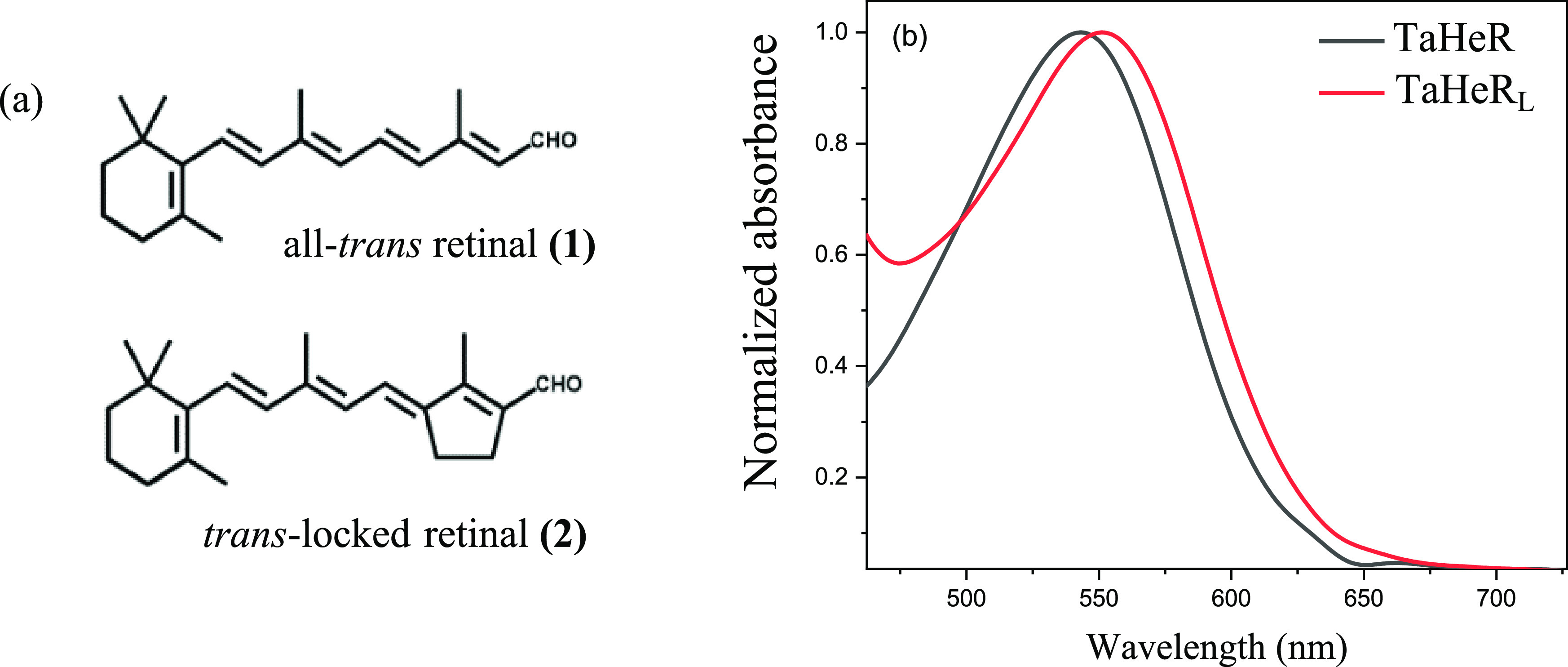
(a) Molecular structure of retinal chromophores. (b) Normalized absorption spectra of the native heliorhodopsin (TaHeR) and the reconstituted artificial pigment (TaHeR_L_).

To monitor protein conformational alterations, we have employed two different reactions: one is the reaction of retinal protonated Schiff base with hydroxylamine forming a retinal oxime, and the other is the thermal denaturation of the protein. The thermal bleaching of retinal proteins was proposed to proceed via the retinal protonated Schiff base hydrolysis, which was suggested to be the rate-determining step.^15^ To explore if retinal double-bond isomerization is a prerequisite for light-induced protein conformational alterations in HeR, experiments were performed on the native pigment as well as on an artificial pigment (TaHeR_L_) derived from a “locked” all-*trans* retinal analogue (**2**-Figure 1a) in which the isomerization around the C_13_=C_14_ double bond is prevented^5^ via a rigid five-membered ring structure (Figure 1a).

### Hydroxylamine Reaction

**Figure 2:**
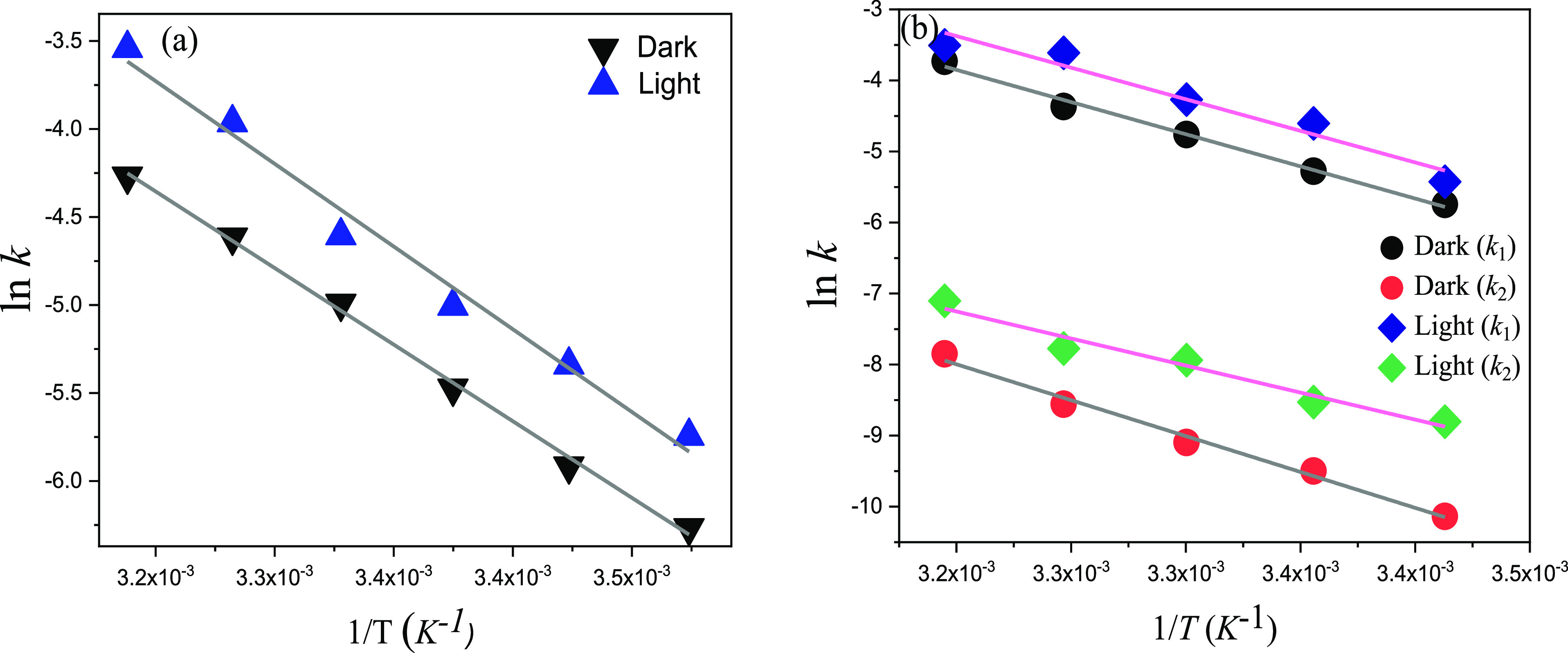
Arrhenius plots of hydroxylamine (0.5 M) reaction with (a) TaHeR and (b) TaHeR_L_ in the absence and presence of light.

The absorption maximum of the artificial locked pigment (TaHeR_L_) is red-shifted from 542 to 552 nm relative to the native TaHeR (Figure 1b). Following the formation of retinal oxime, the characteristic absorption maxima of both TaHeR and TaHeR_L_ decrease accompanied by the formation of a retinal oxime absorption band at 360 nm. The decrease in the pigment band intensity was monitored with time and utilized to estimate the reaction rate. Representative spectra of the hydroxylamine (HA) reaction and the corresponding decay kinetics plot are given in the Supporting Information (Figure S1). The reaction of the native TaHeR with HA is light-accelerated by ∼1.5 times at 27 °C. A light-induced acceleration of the reaction rate was detected also in the artificial TaHeR_L_ pigment derived from the synthetic locked chromophore (**2**). This light-induced rate acceleration in the TaHeR_L_ pigment indicates that the effect is not solely associated with the retinal photoisomerization event. An analysis of the activation energy, the frequency factor, and temperature can shed light on the factors that affect the reaction pathway. Therefore, we evaluated the activation energy and frequency factors of the process for both the native and locked pigments, which are presented in Tables 1 and 2, respectively. The corresponding Arrhenius plots are shown in Figure 2.

**Table 1:** Reaction Rate and Estimated Arrhenius Parameters of the TaHeR–Hydroxylamine (0.5 M) Reaction at Various Temperatures in the Absence and Presence of Light

	dark			light		
temperature (°C)	*k* (s^–1^)	*E*_a_^d^ (kcal·mol^–1^)	*A* (s^–1^)	*k* (s^–1^)	*E*_a_^l^ (kcal·mol^–1^)	*A* (s^–1^)
12	(1.9 ± 0.1) × 10^–3^	14.41 ± 0.1	(2.0 ± 0.07) × 10^8^	(3.2 ± 0.1) × 10^–3^	15.50 ± 0.09	(2.5 ± 0.07) × 10^9^
17	(2.7 ± 0.08) × 10^–3^			(4.8 ± 0.07) × 10^–3^		
22	(4.2 ± 0.06) × 10^–3^			(6.7 ± 0.05) × 10^–3^		
27	(6.8 ± 0.06) × 10^–3^			(1.0 ± 0.05) × 10^–2^		
32	(9.9 ± 0.06) × 10^–3^			(1.9 ± 0.06) × 10^–2^		
37	(1.4 ± 0.1) × 10^–2^			(2.9 ± 0.1) × 10^–2^		

**Table 2:** Reaction Rate and Estimated Arrhenius Parameters of the TaHeR_L_–Hydroxylamine (0.5 M) Reaction at Various Temperatures in the Absence and Presence of Light

dark						
temperature (°C)	*k*_1_ (s^–1^)	*E*_a1_^d^ (kcal·mol^–1^)	*A*_1_^d^ (s^–1^)	*k*_2_ (s^–1^)	*E*_a2_^d^ (kcal·mol^–1^)	*A*_2_^d^ (s^–1^)
20	(3.2 ± 0.01) × 10^–3^	17.98 ± 0.1	(8.1 ± 0.12) × 10^10^	(3.96 ± 0.1) × 10^–5^	20.08 ± 0.1	(3.6 ± 0.12) × 10^10^
25	(5.1 ± 0.09) × 10^–3^			(7.5 ± 0.07) × 10^–5^		
30	(8.5 ± 0.08) × 10^–3^			(1.1 ± 0.08) × 10^–4^		
35	(1.27 ± 0.1) × 10^–2^			(1.9 ± 0.1) × 10^–4^		
40	(2.4 ± 0.1) × 10^–2^			(3.90 ± 0.1) × 10^–4^		

light						
temperature (°C)	*k*_1_ (s^–1^)	*E*_a1_^l^ (kcal·mol^–1^)	*A*_1_^l^ (s^–1^)	*k*_2_ (s^–1^)	*E*_a2_^l^ (kcal·mol^–1^)	*A*_2_^l^ (s^–1^)
20	(4.4 ± 0.07) × 10^–3^	17.66 ± 0.08	(8.0 ± 0.1) × 10^10^	(1.5 ± 0.07) × 10^–4^	15.12 ± 0.08	(2.6 ± 0.1) × 10^7^
25	(1.0 ± 0.06) × 10^–2^			(1.98 ± 0.06) × 10^–4^		
30	(1.4 ± 0.06) × 10^–2^			(3.57 ± 0.06) × 10^–4^		
35	(2.7 ± 0.08) × 10^–2^			(4.2 ± 0.08) × 10^–4^		
40	(3.1 ± 0.09) × 10^–2^			(8.23 ± 0.09) × 10^–4^		

The native pigment–HA reaction exhibits *E*_a_^d^ (activation energy in the dark) < *E*_a_^l^ (activation energy in the presence of light), whereas light irradiation increases the frequency factor by ∼10-fold. Thus, the slightly higher activation energy caused by light is compensated by the higher magnitude of frequency factor reflected in light acceleration. The type 1 rhodopsins previously examined for light acceleration of the HA reaction exhibited a much lower activation energy barrier and a counterbalancing low magnitude of frequency factor, overall making the light process faster in native proteins. In TaHeR_L_, the reaction kinetics is biphasic in both dark and light. The two reaction rates obtained are denoted as *k*_1_ and *k*_2_. One component of the reaction rates is similar in order, whereas the other component is much slower (∼100-fold) than the native pigment both in the dark and light. This difference between the native and artificial pigments can be attributed to the altered retinal binding site imposed by the additional five-membered ring of the synthetic chromophore (**2**). Between the two rate exponents of TaHeR_L_, *E*_a1_^l^ is marginally lower than *E*_a1_^d^, with *A*_1_^l^ ≈ *A*_1_^d^, whereas *E*_a2_^l^ < *E*_a2_^d^, with *A*_2_^l^ < *A*_2_^d^, inducing a faster process in the presence of light. TaHeR adopts a dimeric form at the studied pH (pH 7).^13^ Possibly, the reaction proceeds in a biphasic manner (with a nearly 1:1 contribution of the two rate components) in the dimeric pigment, since the reaction on one segment of the dimeric protein can affect the other one reflected by a slower reaction rate. We assume that in the native pigment, the two components have similar rates and thus a single-exponential decay kinetics is detected. Representative spectra of the HA reaction and the corresponding decay kinetics plot for TaHeR_L_ are given in the Supporting Information (Figure S2).

### Thermal Denaturation

**Figure 3:**
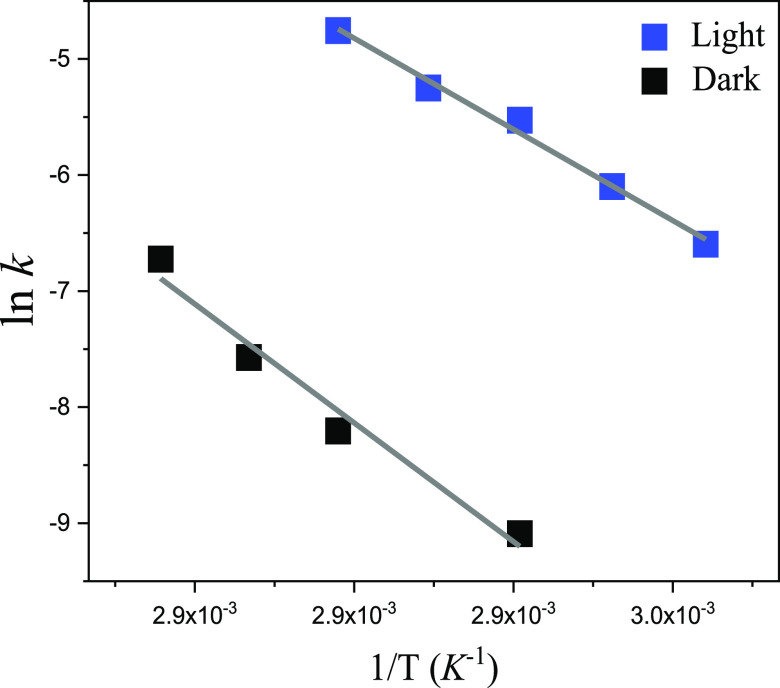
Arrhenius plot of thermal denaturation with TaHeR in the absence and presence of light.

Next, we have checked the thermal denaturation of both pigments. It was proposed that the rate-determining step of the denaturation process of retinal proteins is associated with the hydrolysis of the protonated Schiff base bond, and the process was found to be accelerated by light.^15^ Following the temperature increase of TaHeR, the characteristic pigment absorption band gets reduced accompanied by a formation of retinal absorption band in the 360–370 nm region. A single-exponential decay was found for the native pigment absorbance change. The process is much faster under light relative to the dark reaction (∼35 times faster under light at 67 °C). The activation energy in light *E*_a_^l^ is lower than *E*_a_^d^, with *A*^l^ < *A*^d^ (Table 3). The Arrhenius plot for the TaHeR thermal process is shown in Figure 3. The much higher activation energy in the dark is partly compensated by a higher frequency factor, still making the process significantly faster under light. On the other hand, the rate estimation for TaHeR_L_ was difficult because of the high light scattering arising after a short progress of the reaction. Therefore, we present (Table 4) a comparative account on the percentage of pigment left after a defined time (10 min). The greater extent of pigment bleached after this time clearly indicates that this process is light-accelerated in the locked pigment too. To explore whether this substantially higher light acceleration in the thermal denaturation process as compared to the HA reaction is general for other retinal proteins, we measured the rates of both these reactions in *Gloeobacter* rhodopsin (GR) as a representative of type 1 retinal proteins. However, in GR, the extent of light acceleration in the thermal process was found to be very similar to the HA reaction (Table 5).

**Table 3:** Reaction Rate and Estimated Arrhenius Parameters of the TaHeR Thermal Denaturation Process at Various Temperatures in the Absence and Presence of Light

	dark			light		
temperature (°C)	*k* (s^–1^)	*E*_a_ (kcal·mol^–1^)	*A* (s^–1^)	*k* (s^–1^)	*E*_a_ (kcal·mol^–1^)	*A* (s^–1^)
63		68.00 ± 0.1	(3.35 ± 0.12) × 10^39^	(1.37 ± 0.05) × 10^–3^	52.00 ± 0.1	(1.0 ± 0.1) × 10^31^
65				(2.25 ± 0.06) × 10^–3^		
67	(1.13 ± 0.08) × 10^–4^			(3.99 ± 0.06) × 10^–3^		
69				(5.26 ± 0.07) × 10^–3^		
71	(2.74 ± 0.08) × 10^–4^			(6.83 ± 0.08) × 10^–3^		
73	(5.16 ± 0.07) × 10^–4^					
75	(1.23 ± 0.07) × 10^–3^					

**Table 4:** Percentage of TaHeR_L_ Pigment Remaining after 10 min at Various Temperatures in the Absence and Presence of Light

temperature (°C)	percentage of pigment left (dark)	percentage of pigment left (light)
52	3.5 ± 0.1	15 ± 0.06
55	8.9 ± 0.1	22 ± 0.07
58	13.0 ± 0.1	30 ± 0.07

**Table 5:** Comparison of the Dark/Light Reaction Rate of the Hydroxylamine Reaction and Thermal Denaturation with TaHeR and GR

	hydroxylamine reaction (25 °C)			thermal denaturation		
pigment	dark	light	ratio (light/dark)	dark	light	ratio (light/dark)
TaHeR (native)	(4.2 ± 0.06) × 10^–3^	(6.7 ± 0.05) × 10^–3^	1.6	(2.7 ± 0.08) × 10^–4^ (71 °C)	(6.8 ± 0.08) × 10^–3^ (71 °C)	25
GR	(6.5 ± 0.06) × 10^–4^	(1.5 ± 0.06) × 10^–3^	2.3	(2.0 ± 0.07) × 10^–3^ (70 °C)	(3.0 ± 0.08) × 10^–3^ (70 °C)	1.5

It is evident that the two studied nonphotochemical reactions involving the retinal protonated Schiff base show light-induced rate enhancement, which can result from retinal isomerization and/or protein conformational alterations. The light-induced modified protein accelerates the reactions probably by enhancing the accessibility of the reagent and/or lowering the reaction energy of activation. Strikingly, this light acceleration is observed even with the artificial pigment in which the retinal double-bond isomerization is prevented. An earlier study with bacteriorhodopsin first proposed the involvement of the M photo-intermediate, where a considerable structural change of the protein assists a faster reaction with HA.^16^ Later the L intermediate was suggested to be the species involved in light catalysis rather than M.^17^ Based on theoretical considerations, it was proposed that a large light-induced charge redistribution takes place in the retinal polyene that polarizes the protein and generates intermediate conformational states to stabilize the new protein structure in the excited state.^18,19^ The large induced charge redistribution in the excited state is maintained and even increased during the excited-state lifetime.^20−24^ Later, second harmonic generation (SHG), two-photon spectroscopy, optical rectification, and terahertz radiation studies demonstrated that indeed a large light-induced dipole is developed in bacteriorhodopsin following light absorption.^25−28^ Optical rectification studies estimated that the projection of the induced dipole on the membrane normal is 11 D, corresponding to the displacement of a full charge over approximately half the length of the retinal chromophore.^28^ SHG measurements were further used to propose that the large induced dipole developed in the retinal chromophore is due to retinal protein–tryptophan interaction, since tryptophan is capable of stabilizing a positive charge due to its high polarizability.^27^ Ultrafast spectroscopy indicated that tryptophan responds to the retinal excited state and its absorption decreases within the first 200 fs of the retinal excited-state lifetime.^29^

The atomic force sensing (AFS) method detected light-induced protein structural alterations in both native bacteriorhodopsin and artificial pigment derived from a nonisomerizable retinal analogue.^7^ Moreover, the AFS signal kinetics of native bacteriorhodopsin contained components that were not correlated with the known photointermediates, indicating the involvement of other spectrally silent intermediates.^7^ Thus, it was suggested that the light-induced charge delocalization in the retinal excited state polarizes the protein environment, thereby inducing perturbation in the protein conformation, which persists over an extended microseconds time range.

Data on the excited-state dipole of HeR is not available but it is conceivable that the retinal chromophore embedded in the TaHeR opsin binding site acquires an analogous light-induced large transient dipole in the initial excited state. We note that the topology of the retinal protonated Schiff base in TaHeR is opposite to other retinal proteins and the protonated Schiff base proton is facing the intracellular protein side in contrast to other retinal proteins. The residues in bacteriorhodopsin that were suggested to participate in the stabilization of the large excited-state dipole are Trp182, Trp138, and Trp189. These residues are replaced in TaHeR by Phe203, Tyr164, and Met210, respectively.^13^ The only tryptophan residue present near the retinal binding site in TaHeR is Trp106, located within a distance of 6.3 Å from the β-ionone ring.^13^ The other aromatic residues present around the retinal binding site (within 4.5 Å) are Phe203, Phe206, Tyr109, and Tyr164.^13^ Thus, TaHeR lacks the conserved tryptophan residues detected in the type 1 family. However, the presence of phenylalanine and tyrosine residues can stabilize the retinal excited-state charge redistribution and a large light-induced excited-state dipole. Thus, we propose that light absorption by the retinal chromophore in TaHeR will polarize the protein to trigger protein conformational changes. Therefore, it is plausible that in HeRs, light acceleration of both the examined reactions involving the locked retinal chromophore indicates protein conformation changes that take place without the retinal isomerization. The lifetime of TaHeR excited state is in the sub-picosecond region similar to other retinal proteins.^30^ The excited-state lifetime of the locked pigment was studied for the bacteriorhodopsin case, and it was found to be ∼20 ps.^31^ It is conceivable that the excited-state lifetime of the locked TaHeR_L_ is of the similar order. Chemical reactions with hydroxylamine and the denaturation process are much slower, and thus, probably they take place after the relaxation of the excited state to the ground state, on a timescale longer than microseconds. Therefore, we suggest that a relatively long-lived protein structural change is induced in the locked pigment as a response to the retinal excited-state charge redistribution, leading to acceleration of the reactions.

Analysis of the kinetic parameters of the HA reaction in TaHeR and other type 1 rhodopsins studied so far indicates an inverse pattern of the activation energy and the frequency factor modulation shifting from dark to light adapted reaction. In bacteriorhodopsin and the other studied rhodopsins, illumination leads to a much lowered activation energy barrier along with a partial counterbalancing contribution from the reduced frequency factor.^8−11^ In TaHeR, however, the light acceleration is due to the higher frequency factor that makes the reaction faster by overcoming the relatively unfavorable activation energy gap. This indicates that effective collisions between the reactants are increased in the TaHeR light reaction, whereas they are decreased significantly in the other studied retinal proteins. This is likely because of the differences in the retinal binding pocket structure and the resulting distinct geometry of the protonated Schiff base, as was also suggested by the resonance Raman study.^14^ It is possible that due to this somewhat different geometrical arrangement of the retinal protonated Schiff base in the binding pocket, the accessibility of the retinal site for HA following light-activated protein conformational changes is different in TaHeR relative to type 1 rhodopsins. On the other hand, light acceleration accompanies a reduced activation energy and an increased frequency factor in TaHeR_L_, unlike that in native TaHeR. This also can be ascribed to the geometrical difference of the rigid-ring-structured chromophore in the binding pocket as compared to the native retinal.

The thermal denaturation at a higher temperature is a consequence of gradual unfolding of the protein secondary structure,^32^ thus allowing more water molecules to penetrate the retinal binding site or accelerate the water-protonated Schiff base reaction. At an elevated temperature, the opening of the protein fold (at least partially) can respond differently during the conformational perturbation and availability of the retinal pocket for hydrolysis to allow the light reaction through a different pathway. This can explain the contrary pattern of Arrhenius parameters and the significantly faster light reaction rate as compared to the retinal oxime formation process (the light/dark reaction rate ratio is ∼1.5 for HA reaction and ∼35 for the thermal process at two specific temperatures). Moreover, as mentioned above, TaHeR shows a comparatively enhanced light effect of thermal denaturation relative to the HA reaction, which is not observed in another type of rhodopsin GR (light effect was nearly the same for both the processes in GR). This observation emphasizes the unique protein response of HeR.

It is very likely that the light-induced dipole developed in the retinal excited state triggers protein conformational alterations that accelerated the chemical reaction. This proposal is supported by the recent time-resolved X-ray structure that detected protein conformation changes during the lifetime of the retinal excited state in bacteriorhodopsin.^33,34^ Still, it is possible that the chemical reaction rate acceleration is caused by heat dissipation following the formation of the retinal excited state rather than protein polarization and response to the retinal light-induced dipole. This possibility is highly unlikely based on the work carried out on BR in which the retinal protonated Schiff base protein linkage was reduced with sodium borohydride.^6^ The reduction reaction led to a symmetric polyene covalently bound to the protein in which significant electronic charge redistribution following light absorption can be excluded. This pigment did not show any light-catalyzed reaction, although heat dissipation can take place. Thus, the lack of light catalysis in the reaction of the symmetric chromophore and its presence in asymmetric systems excludes the possibility that protein structural changes are induced by excess light energy dissipated as heat. This observation is consistent with the suggested mechanism that the catalytic conformational alterations are associated with charge redistribution following light absorption.

However, an alternative mechanism for the light acceleration of the chemical reaction cannot be completely excluded. Recently, fast dynamic (picosecond to nanosecond timescale) effects of functionally relevant vibronic motions have been proposed to couple with the chemical process in enzymatic catalysis. For example, local collective domain motion ranging in picoseconds to seconds and their interplay was suggested to assist the slow opening of the active-site lid in adenylate kinase.^35^ The criterion of an effective promoting vibration is its coupling with the progression of the reaction coordinate.^35^ Another example is based on a photosynthetic microorganism system, where a primary charge-separation dynamics gets facilitated upon coupling to a coherent nuclear motion.^36^ It is possible that due to the illumination of HeR, the retinal chromophore couples to specific protein vibrations, with their energy matching the chemical reaction coordinate between the reagent and the retinal protonated Schiff base, which in turn enhances the reaction rate.

It can be concluded that despite its special geometrical chromophore arrangement and structural diversity, HeR undergoes light-induced protein conformational fluctuations even with a nonisomerizable retinal chromophore, which is reflected in the rate enhancement of retinal Schiff base linkage reactions. However, here we have noted that the light-induced response of the TaHeR protein is somewhat different as the reaction parameters are modified in a contrasting manner to that of other retinal proteins. Importantly, blocking the double-bond isomerization does not block the protein conformational alterations but prevents the biological activity. Namely, it was shown in bacteriorhodopsin that formation of the M photochemical intermediate and deprotonation of the retinal protonated Schiff base are required for the proton-pumping process. Future studies should shed light on the role played by protein conformational alterations that are triggered by the retinal excited state and their coordination with the changes induced by the retinal isomerization. In this respect, we note that previous studies indicated that the elimination of the retinal dipole and protein conformational alteration induced by the retinal excited state prevented retinal isomerization and initiation of the photocycle.^37^ Therefore, it is possible that polarization of the protein and initiation of protein changes in the excited state assist and make an efficient retinal double-bond isomerization possible.

## Supporting Information Available

Experimental methods; absorption spectrum of TaHeR–hydroxylamine reaction and the corresponding absorbance decay plot with time; absorption spectrum of TaHeR_L_–hydroxylamine reaction and the corresponding absorbance decay plot with time; and absorption spectrum of TaHeR thermal denaturation and the corresponding absorbance decay plot with time (PDF)
