# Supplementary material for: Light-Induced Conformational Alterations in Heliorhodopsin Triggered by the Retinal Excited State
Source: J Phys Chem B. 2021 Aug 3;125(31):8797–804. doi: 10.1021/acs.jpcb.1c04551 (PMC8389987; doi:10.1021/acs.jpcb.1c04551)
Supplement: Supplementary file 1 — jp1c04551_si_001.pdf [file jp1c04551_si_001.pdf]

Supporting Information

Light-Induced Conformational Alterations in Heliorhodopsin Triggered by the Retinal Excited State

Ishita Das,[1] Alina Pushkarev[2], and Mordechai Sheves*[1]

[1]Weizmann Institute of Science, 7610001 Rehovot, Israel

[2]Faculty of Biology, Technion–Israel Institute of Technology, Haifa 3200003, Israel

* e-mail: mudi.sheves@weizmann.ac.il

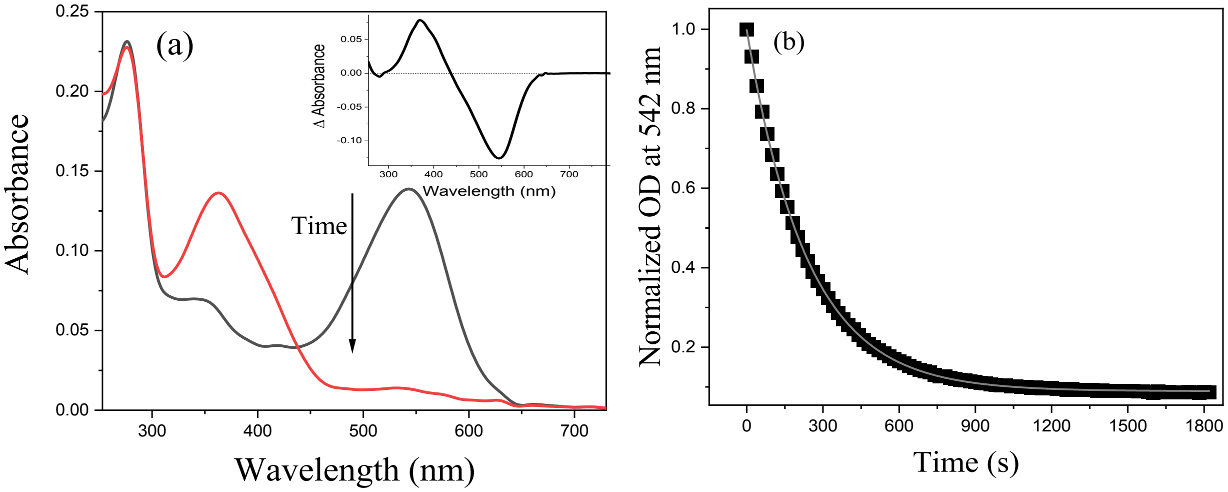

Figure S1. (a) Absorption spectra of TaHeR-Hydroxylamine reaction with time, at 22°C, in dark. Inset figure represents the corresponding difference absorption spectrum. (b) Corresponding change of absorption intensity (normalized) at the absorption maxima 542 nm of TaHeR with progression of the reaction.

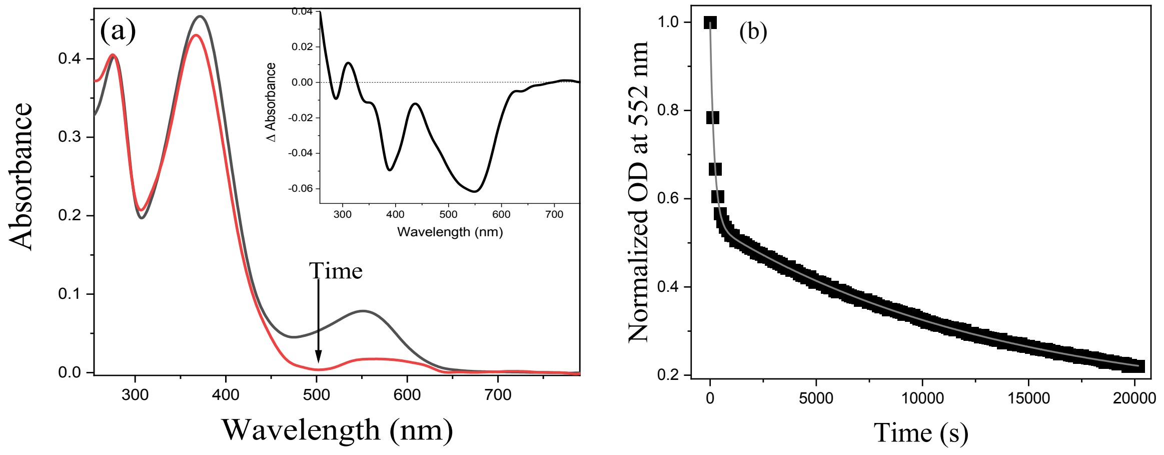

Figure S2. (a) Absorption spectra of $TaHeR_L$-Hydroxylamine reaction with time, at 25°C, in dark. Inset figure represents the corresponding difference absorption spectrum. (b) Corresponding change in absorption intensity (normalized) at the absorption maxima 552 nm of $TaHeR_L$ with progression of reaction.

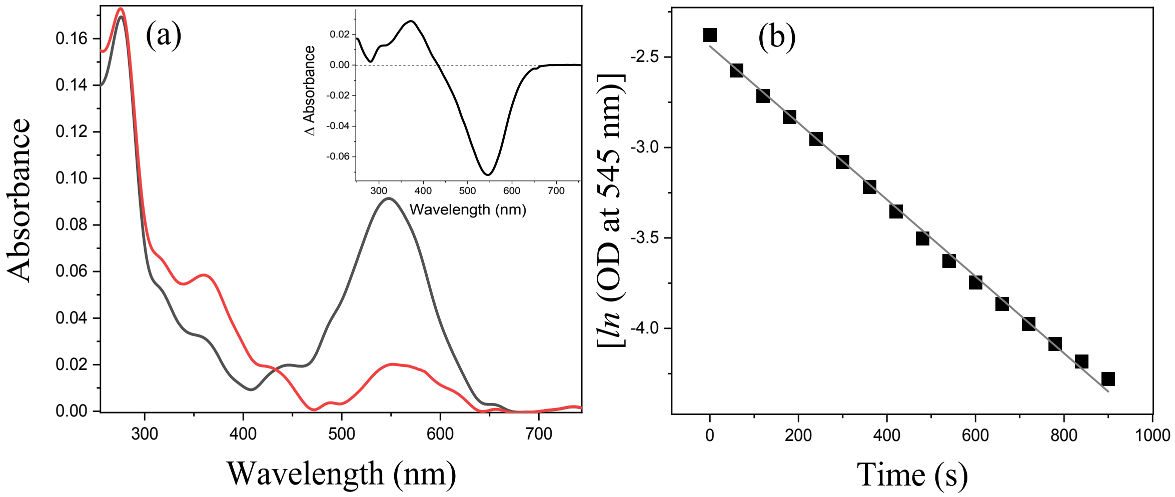

Figure S3. (a) Absorption spectra of TaHeR thermal denaturation with time, at 65°C, in presence of light. Inset figure represents the corresponding difference absorption spectrum. (b) Corresponding change in absorption intensity at 545 nm of TaHeR with progression of the denaturation process.
